# Harnessing microbial antigens as cancer antigens: a promising avenue for cancer immunotherapy

**DOI:** 10.3389/fimmu.2024.1411490

**Published:** 2024-07-30

**Authors:** Tao Zhang, Xilong Zhang, Jianquan Chen, Xiuwei Zhang, Yunlei Zhang

**Affiliations:** ^1^ Department of Respiratory and Critical Care Medicine, The Affiliated Jiangning Hospital of Nanjing Medical University, Nanjing, China; ^2^ Department of Biomedical Engineering, School of Biomedical Engineering and Informatics, Nanjing Medical University, Nanjing, China; ^3^ Department of Burns and Plastic Surgery, First People’s Hospital of Xuzhou City, Xuzhou, China; ^4^ Central Laboratory, Translational Medicine Research Center, The Affiliated Jiangning Hospital of Nanjing Medical University, Nanjing, China

**Keywords:** microbial antigen peptide, tumor antigen, microorganism, immunotherapy, molecular mimicry

## Abstract

Immunotherapy has revolutionized cancer treatment by leveraging the immune system’s innate capabilities to combat malignancies. Despite the promise of tumor antigens in stimulating anti-tumor immune responses, their clinical utility is hampered by limitations in eliciting robust and durable immune reactions, exacerbated by tumor heterogeneity and immune evasion mechanisms. Recent insights into the immunogenic properties of host homologous microbial antigens have sparked interest in their potential for augmenting anti-tumor immunity while minimizing off-target effects. This review explores the therapeutic potential of microbial antigen peptides in tumor immunotherapy, beginning with an overview of tumor antigens and their challenges in clinical translation. We further explore the intricate relationship between microorganisms and tumor development, elucidating the concept of molecular mimicry and its implications for immune recognition of tumor-associated antigens. Finally, we discuss methodologies for identifying and characterizing microbial antigen peptides, highlighting their immunogenicity and prospects for therapeutic application.

## Highlights

- Microbial peptides mimicking tumor antigens.
- Elevated immunogenicity of host-homologous microbial antigens surpassing tumor-associated counterparts.
- Bacterial antigenic peptides as promising candidates for tumor vaccines.
- Exploring host-homologous microbial antigens through innovative strategies.

## 1 Introduction

Immunotherapy has ushered in a new era in cancer treatment, offering tailored strategies to harness the immune system’s intrinsic capabilities in combating malignancies (1, 2). Central to the success of immunotherapy are tumor antigens, which serve as crucial targets for immune recognition and subsequent elimination of cancerous cells while sparing normal tissues from collateral damage (3). However, despite the promise of cancer antigens in immunotherapeutic interventions, their clinical efficacy remains limited due to various challenges, including the constrained repertoire of antigens capable of eliciting robust and enduring anti-tumor immune responses (4). Moreover, the intricate landscape of tumor heterogeneity, characterized by variations in antigen expression profiles among patients and within individual tumors, poses a formidable barrier to the development of effective immunotherapeutic approaches (5). Furthermore, cancer cells employ a repertoire of immune evasion mechanisms, such as antigen downregulation and alteration of antigen processing pathways, further hampering immune recognition and compromising therapeutic outcomes (6). Consequently, there is a critical need to identify and validate novel tumor antigens through comprehensive profiling of tumor cells and the surrounding microenvironment, with the aim of uncovering immunogenic targets accessible to immune effectors. Recent advancements have highlighted the unique immunogenic properties of host homologous microbial antigens, which offer the potential to elicit robust immune responses against tumors while minimizing off-target effects on normal tissues (7). For instance, Wells et al. proposed the concept of pathogenic peptide homology as a strategy for evaluating neoantigen immunogenicity, suggesting that comparisons with exogenous microbial peptides could facilitate the identification of neoantigens for cancer immunotherapy (8, 9). In this review, we aim to explore the therapeutic potential of homologous microbial antigen peptides in tumor immunotherapy. We commence by providing an overview of tumor antigens and their characteristics, followed by an examination of the influence of microorganisms on tumor development and treatment. Subsequently, we summary the concept of molecular mimicry, elucidating the structural similarities between microbial antigens and tumor-associated antigens. Finally, we present a comprehensive overview of methods for obtaining microbial antigen peptides, discussing their immunogenicity and potential applications in cancer immunotherapy.

## 2 Tumor specific antigen and tumor associated antigen

Tumor antigens encompass two primary categories: tumor-specific antigens (TSAs), also known as neoantigens, and tumor-associated antigens (TAAs). TSAs originate from nonsynonymous mutations or other genetic alterations, leading to the production of mutant peptides exclusively expressed in tumor cells (10). In contrast, noncanonical neoepitopes arise from various mechanisms such as alternative splicing, post-translational modifications, RNA editing, and aberrant mRNA translation (9). These tumor neoantigens, as immunogenic non-autoantigens, demonstrate specificity in their expression by tumor cells, thereby eliciting highly targeted immune responses against the malignancy (9, 11). Consequently, neoantigens exhibit two defining characteristics: exclusive presence on tumor cells and the capacity to evade central immune tolerance mechanisms, effectively stimulating T cell responses. For example, a recently identified melanoma epitope has been recognized by CD4^+^ T cells, with a neoantigen vaccine predominantly activating this subset of immune cells (12, 13). Crucial to targeted tumor therapy leveraging neoantigens is the identification of those possessing robust immunogenicity capable of eliciting potent T cell responses (9). Notably, several neoantigens share sequence homology with peptides derived from microbial sources, with accumulating evidence indicating their heightened immunogenic potential compared to pathogenic antigen homologs and non-homologous neoantigens (14, 15).

The personalized nature of neoantigens presents a significant hurdle for their widespread application, as they are uniquely tailored to individual patients and thus impractical for large-scale use (13, 16, 17). Furthermore, the dynamic mutation landscape of tumors can lead to the continuous generation of novel neoantigens, allowing tumors to evade immune surveillance (10). In contrast, TAAs exhibit broader relevance across various malignancies, as they are self-antigens overexpressed in tumor cells while also shared with normal tissues. Consequently, TAAs represent promising targets for the development of cancer vaccines on a larger scale (10). For instance, the melanoma-associated antigen (MAGE) gene family-encoded antigens MZ2-E and MZ2-D have been identified as elicitors of anti-tumor immune responses mediated by cytotoxic T cells since the 1990s (18, 19). However, the ubiquitous expression of TAAs in normal tissues poses a challenge, potentially inducing immune tolerance and impeding effective anti-tumor immune responses (20, 21). Moreover, TAA-specific T cells may inadvertently target corresponding normal tissues, precipitating autoimmune reactions (22, 23). In light of these challenges, Tagliamonte et al. proposed that exploiting the homology between TAAs and microbial antigens may offer a promising strategy to enhance the efficacy of cancer immunotherapy (10).

## 3 How intratumoral microorganisms shape the immune landscape within tumors

### 3.1 Reciprocity between intratumoral microorganisms and tumor cells

The term “microbiome in tumors” encompasses the diverse microbial communities residing within tumor tissues or their immediate surroundings, comprising bacteria, fungi, viruses, and other microorganisms (24). These entities can inhabit tumor cells directly or parasitize tissues, blood, or adjacent body fluids. Emerging research indicates that various types of tumors harbor distinct microbial compositions, suggesting a close association between microbial diversity and tumor types (25, 26). In the context of breast cancer, for instance, the presence of specific microorganisms within breast cancer tissues has been implicated in facilitating tumor metastasis, highlighting the functional significance of the tumor microbiome (27). Moreover, the spatial organization of microflora within tumors reflects a non-random distribution pattern, occupying discrete niches that modulate immune responses and epithelial cell functions. Interactions between these microorganisms and tumor cells can promote cancer progression through various mechanisms, including direct stimulation of cell proliferation, suppression of immune responses, and modulation of the tumor microenvironment (28).

The influence mechanisms of tumor-associated microorganisms on tumor development have garnered considerable attention. On one hand, these microorganisms may induce DNA damage, manipulate host gene expression, or interfere with signaling pathways, thereby promoting tumorigenesis (29, 30). Alternatively, they can contribute to tumor progression by inducing immunosuppressive states, fostering pro-inflammatory responses, and activating carcinogenic pathways (31). Furthermore, microbial metabolites may interfere with chemotherapy efficacy, leading to drug resistance (32). Conversely, the presence of microorganisms within tumors may induce inhibitory effects on tumor progression. Studies suggest that the diversity of tumor-associated microorganisms correlates with the survival outcomes of pancreatic cancer patients, with interplay observed between the microbial compositions of pancreatic tumors and the intestinal microbiota (33). Notably, certain microbial compounds produced within tumors may enhance anti-tumor immunity, though their precise origins remain unclear (34). Moreover, the tumor microbiome can influence the efficacy of immunotherapy by modulating immune responses and altering tumor immunogenicity (31). In addition, bacterial influences on tumor outcomes extend to transcriptional pathways that directly impact inflammation, tumor growth, and immune cell infiltration, underscoring the multifaceted nature of microbial contributions to cancer pathogenesis (28, 33).

### 3.2 Unveiling the mechanisms: microbial-mediated immune regulation

**Figure 1:**
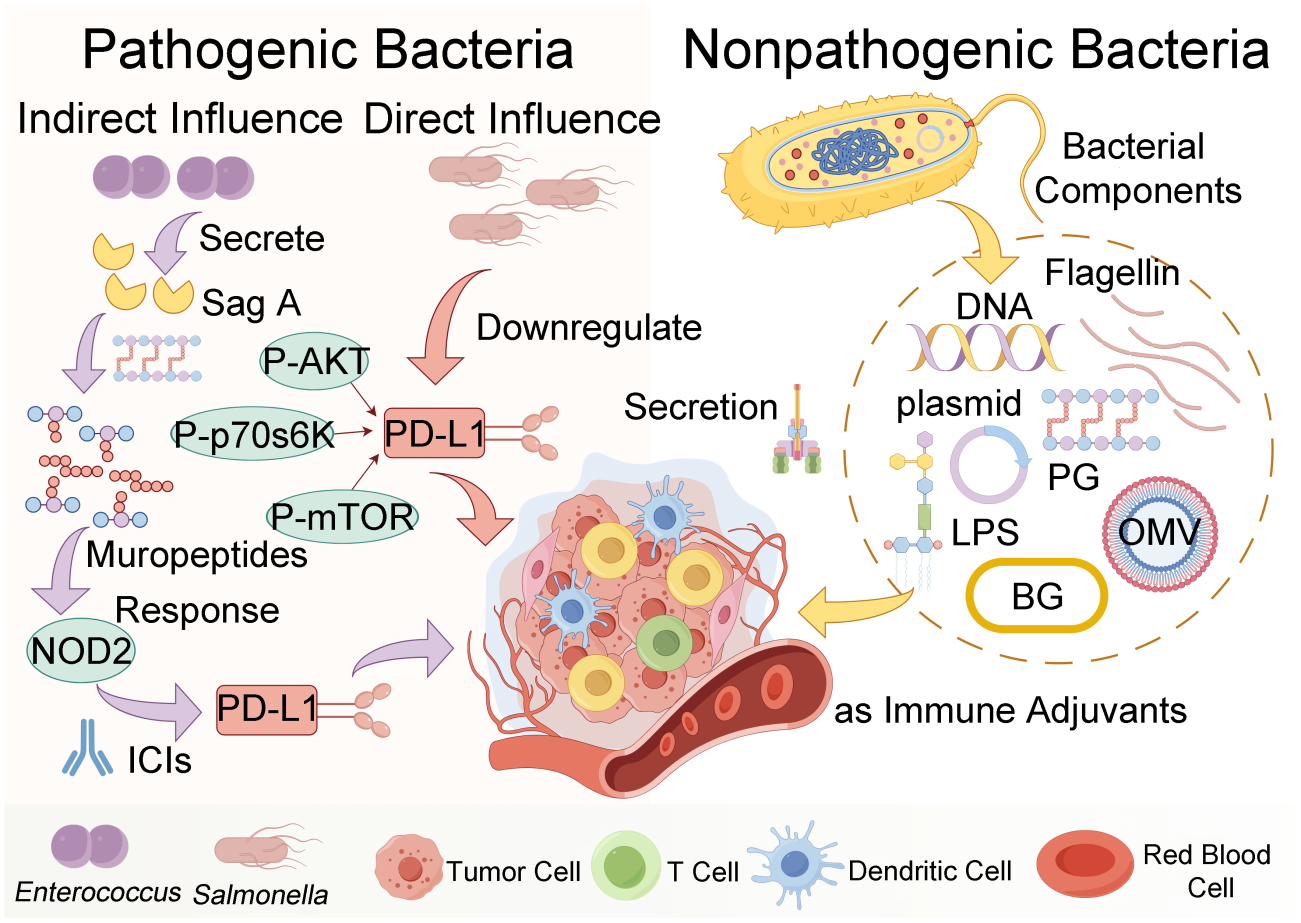
The figure illustrates the mechanisms by which pathogenic or nonpathogenic bacteria influence the tumor microenvironment and bolster anti-tumor immune responses. Key components such as peptidoglycan (PG), lipopolysaccharide (LPS), outer membrane vesicles (OMV), and bacterial ghosts (BG) interact with immune cells and signaling pathways, including secreted antigen A (Sag A), immune checkpoint inhibitors (ICIs), nucleotide-binding oligomerization domain 2 (NOD2) and programmed death ligand-1 (PD-L1), phospho-protein kinase B (P-AKT), phospho-mammalian targets of rapamycin (P-mTOR), and phospho-p70 ribosomal s6 kinase (P-p70s6K), to achieve these effects (created by Figdraw).

Immunotherapy for tumors encompasses a spectrum of modalities, including molecular targeted therapy utilizing monoclonal antibodies, immune checkpoint inhibitor (ICI) therapy, adoptive cellular immunotherapy such as chimeric antigen receptor T cell (CAR-T) therapy, cytokine therapy involving interleukin-2 (IL-2) and interferon-gamma (IFN-γ), and tumor vaccines (35, 36). Among these approaches, ICI therapy stands out as a mature and extensively studied modality. Its primary mechanism involves the upregulation of T cell-mediated immune killing by targeting co-suppressor molecules, notably programmed death protein-1/programmed death ligand-1 (PD-1/PD-L1), thereby bolstering endogenous host immunity and counteracting tumor immune evasion (37, 38). Despite the clinical approval of several ICIs for cancer treatment, challenges persist, including low response rates and the emergence of drug resistance in many cases (6, 39–43). The intricate interplay between intestinal microorganisms and host immunity can indirectly influence cancer patients’ responses to ICIs (**Figure 1**) (44, 45). For instance, active *Enterococcus* secreted antigen A (Sag A), may enhance host immunity by interacting with nucleotide-binding oligomerization domain 2 (NOD2), potentially augmenting the anti-tumor effects of anti-PD-L1 therapy (46). In the realm of anti-tumor immunity, microbial communities exert regulatory control over metabolites through the stimulation of pathogen-related molecular patterns, thereby contributing to the formation of an adaptive immune repertoire by inducing cross-reactive T cell responses (44, 47–49).

Indeed, bacteria serve as natural immune antigens capable of activating immune cells to mount anti-tumor responses (**Figure 1**). Studies have demonstrated the potential of *Salmonella* in inducing direct tumor cell death and modulating the tumor microenvironment via immune-mediated mechanisms, thereby promoting tumor suppression (50, 51). For instance, *Salmonella* has been shown to downregulate the expression of programmed death-ligand 1 (PD-L1) in tumors, thus altering tumor immune tolerance (52). Moreover, recent research by Gao et al. has unveiled the therapeutic potential of a mixture of four Clostridium strains, designated CC4, in enhancing programmed cell death protein 1 (PD-1) therapy. This innovative approach holds promise as an independent immunotherapeutic strategy for melanoma and colorectal cancer (53).

Bacteria also possess the potential to modulate immune cells as therapeutic agents, as depicted in **Figure 1**. Non-pathogenic bacteria can interact with phagocytes and regulate various immune cells within the tumor microenvironment, including macrophages, dendritic cells, and neutrophils (54, 55). The early induction of monocyte-derived dendritic cells (DCs) serves as a hallmark of successful activation of the tumor immune response (56). Bacterial factors are frequently utilized as immune adjuvants in DC-based anti-tumor treatments, exemplified by the use of 50S ribosomal protein from *Mycobacterium tuberculosis* and w-*Mycobacterium* acid mutase TBCM, as well as FcγR secreted by *Staphylococcus aureus* (56, 57). NK cells, belonging to the innate lymphocyte (ILC) family, exhibit versatile immune functions within tumors (58). Bacteria such as *Salmonella* and *Mycobacteria* have been shown to impede the progression of numerous cancers in an NK cell-dependent manner (59, 60). This effect is mediated by the stimulation of NK cells to produce IFN-γ, which orchestrates the accumulation, activation, and cytotoxicity of NK cells, thereby restraining tumor metastasis (61). A large number of preclinical evidence highlights the reliance of bacteria on T cells to instigate anti-tumor adaptive immune responses (47). For instance, a vaccine targeting *Helicobacter pylori* activates CD3^+^ T cell immune responses and suppresses the growth of germinal center (GC) cells. Consequently, the T cell immunity induced by bacteria cross-reacts with cancer cell major histocompatibility complex class I (MHC-I) restriction antigens, underscoring the role of microbial-specific T cells in bolstering anti-tumor immune responses (62, 63).

## 4 Use of microbial peptides as cancer neoantigens for immunotherapy

### 4.1 Molecular mimicry of microbial peptides to human antigens

The concept of molecular mimicry, also known as antigen mimicry, was first proposed in 1964 to elucidate the phenomenon wherein infectious pathogens display structural similarities to antigens expressed by human cells, enabling them to evade host immune responses and establish more invasive infections (64). Molecular mimicry not only facilitates pathogen immune evasion but can also precipitate autoimmune diseases. For instance, the epitope SVYRYYGL (SVY) expressed in *Bifidobacterium brevis* bears resemblance to the epitope SIYRYYGL (SIY) found in tumors, enabling SVY-specific T cells to recognize and suppress tumor growth (65). Similarly, the antigenic epitope tail tape measure protein 1 (TMP1) within the genome of the bacteriophage *Enterococcus hirae* shares similarities with the tumor antigen proteasome β subunit 4 (PSMB4). This molecular mimicry can activate CD8^+^ T cells and enhance the efficacy of PD-1 blocking therapy (62). Numerous prior studies, as highlighted by Ting et al., have established a link between various autoimmune diseases and molecular mimicry involving antigens derived from microorganisms (66, 67). Given the vast proteomic diversity of the intestinal microbiome, it is conceivable that additional microbial antigens exhibiting high homology with cancer antigens may be identified (68).

### 4.2 Identification of microbial antigens as cancer neoantigens for cancer therapy

Zitvogel et al. proposed two hypotheses to elucidate the microbial mechanisms underlying tumor immune surveillance (44). Alongside the non-antigen pathway, which involves microorganisms modulating T cell anti-tumor activity through metabolites derived from pathogen-associated molecular patterns (PAMPs), an antigen pathway exists (69). In this antigen pathway, microbial antigens bearing high similarity to tumor antigens activate specific anti-tumor T cells, thereby influencing the immune system and inducing T cell cross-reactivity (44, 70).

Microbial antigens (Ags) encompass surface or internal molecules of microorganisms capable of eliciting immune responses. These substances include various bacterial components such as flagellin, lipopolysaccharides (LPS), cyclic dinucleotides, and bacterial metabolites including toxins, monophosphate lipids, and transforming growth factors (55). Of particular interest is the principle of microbial resistance, which underscores the recognition of tumor-associated bacterial peptides sharing homology with human peptides. Research by Kalaora et al. demonstrated that polypeptides produced by bacteria infiltrating tumor cells can be displayed on the surface of tumor cells, subsequently recognized by the human immune system (63). Through the utilization of 16S rRNA gene sequencing and human leukocyte antigen (HLA) peptidomics, they identified a peptide library derived from intracellular bacteria, revealing 248 HLA-I peptides and 35 HLA-II peptides from 41 bacterial species across 17 melanoma metastases from 9 patients. These bacterial polypeptides constitute a novel class of tumor antigens previously unrecognized (63). Compared to human HLA peptides, bacterial peptides exhibit noticeably higher hydrophobicity, rendering them more conducive to antigen presentation and T cell recognition (63, 71, 72). In addition, clustering analysis of HLA-I peptides indicates that bacterial peptides typically range between 8 and 13 amino acids in length, exhibiting relatively low complexity. This structural simplicity may facilitate immune recognition and subsequent presentation to immune cells for eliciting immune responses (63). It is noteworthy that the selection of bacterial species for immunotherapy should favor those with known negative impacts on host responses and immune system function (63), rather than “protective” bacteria (73, 74).

Therefore, we emphasize that the presence of cancer cells and bacteria within the tumor microenvironment may serve as a novel source of epitopes for cancer immunotherapy. The advantages of utilizing these bacterial peptides are manifold: Firstly, neoantigens bearing homology to microbial peptides exhibit heightened immunogenicity compared to non-homologous antigens (9); Secondly, from a host perspective, bacterial peptides are foreign to the host organism, potentially eliciting a more robust immune response (75); Furthermore, microbial molecular mimicry can impede tumor growth by eliciting T cell cross-reactivity (9); Lastly, antigens sharing homology with pathogenic bacteria can be efficiently expressed within bacterial vectors, offering significant convenience for the development of tumor vaccines (76).

### 4.3 Methods and tools for predicting microbial antigen peptides

**Figure 2:**
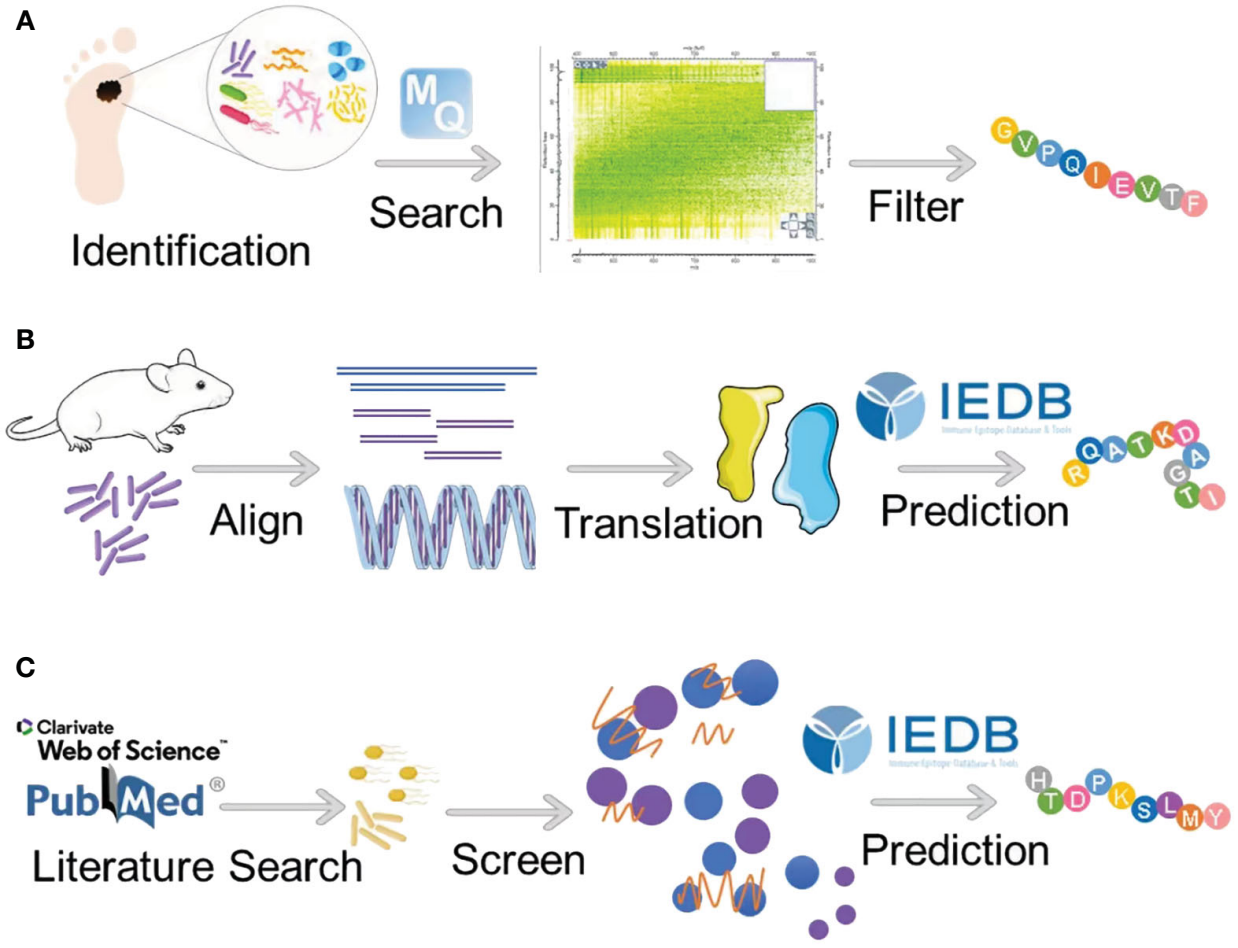
Strategies for identifying microbial antigens for tumor immunotherapy. **(A)** Kalaora et al. used 16S rRNA sequencing to identify bacterial species within tumors and employed bioinformatics to match HLA peptide groups from bacterial and human proteomes, thereby obtaining a filtered set of peptides. **(B)** Wang et al. performed a comparative genomic analysis between *F. nucleatum* and mice, identifying homologous epitopes as potential immunotherapy targets. **(C)** Another strategy involves a literature search to identify tumor-associated pathogenic bacteria, followed by bioinformatics screening for shared amino acid sequences between these bacteria and humans.

In the paper by Naghavian et al., the authors investigated the potential activation of microbial peptides on tumor-infiltrating lymphocytes in glioblastoma, with the objective of elucidating whether microbial antigens could augment immunoreactivity against glioblastomas (77). Their experimental approach focused on assessing immune peptides that bind to HLA class II bacterial peptides in glioblastoma patients. Meanwhile, Kalaora et al. employed 16S rRNA sequencing to identify bacterial species within corresponding tumors. Subsequently, they conducted an analysis of the proteome of the identified bacterial species alongside the human proteome to extract relevant HLA peptide groups, thereby deriving qualified filter peptides (refer to **Figure 2A**). Notably, a key step in their analysis involved comparing the HLA peptide repertoire with the proteome of the corresponding bacteria (63). Furthermore, in the study conducted by Wang et al., a comparative analysis between the genome of *Fusobacterium nucleatum* (*F. nucleatum)* and the mouse genome unveiled the presence of neoantigens originating from bacteria (78). These neoantigens were observed to lack sequence similarity with mice, as well as homologous epitopes sharing similarity between bacteria and mice (see **Figure 2B**).

In this review, we present a method akin to those employed by Kalaora et al. and Wang et al. for the identification of humanized bacterial antigen peptides (63, 78). Our approach begins by compiling a comprehensive list of previously reported pathogenic bacteria known to be associated with tumors. Subsequently, utilizing sophisticated bioinformatics tools, we conduct sequence analyses to identify amino acid sequences within these bacteria that exhibit a high degree of similarity to those present in the human proteome. Following sequence identification, the identified amino acid sequences are subjected to rigorous computational analysis within the Immune Epitope Database (IEDB). Leveraging advanced algorithms such as NetMHCPan, we predict the potential for T cell major MHC-I antigen presentation. The final step of our method involves the meticulous selection of bacterial antigen peptides based on their robust predictive scores and strong affinity for MHC binding. This rigorous selection process ensures the prioritization of peptides with the highest likelihood of eliciting potent T cell responses against tumor cells (**Figure 2C**).

Researchers have developed numerous readily accessible software or tools similar to NetMHCpan for predicting antigenic peptides currently (**Table 1**) (79–88). For instance, MHCpred, based on machine learning algorithms, can rapidly and accurately identify potential MHC-binding peptides by predicting the affinity between peptides and MHC molecules (81). While software such as MaxQuant is not specifically designed for antigen peptide prediction, it aids in the identification of potential antigenic peptides through the analysis of proteomic data (87). MHC molecules consist of two main variants, MHC-I and MHC-II. MHC-I predominantly binds peptides derived from intracellular proteins, while MHC-II mainly interacts with peptides originating from extracellular proteins. Both MHC systems function by presenting non-self protein-derived peptides to T cells, thereby regulating responses against foreign entities (89). The binding between MHC and antigenic peptides represents the most selective step in the antigen presentation pathway, highlighting the significance of predicting peptide-MHC interactions for anticipating the specificity of T cell immune responses (80). In addition, MHC-I molecules bind peptides typically ranging from 8 to 10 amino acid residues, with the ability to accommodate longer peptide ligands (90–92). MHC-II molecules bind peptides of slightly larger average size, usually in the range of 10 to 15 amino acid residues, but can also interact with much longer amino acid sequences (93). In experimental practice, we can adjust and choose appropriate parameters within these software or tools based on the characteristics of MHC molecules to predict antigenic peptides effectively.

**Table 1:** The software or tools to predict antigenic peptides.

Software	Principle	Function	Type	Reference
IEDB	Containing information on immune epitopes, the molecular targets of adaptive immune responses	Including tools for predicting peptide immunogenicity, such as MHC I/II binding prediction and T-cell epitope prediction	Database	(79)
NetMHCpan	Using artificial neural networks (ANN) and binding affinity and mass spectrometry eluted ligand data for training	Predicting the binding of peptides to known sequences of MHC molecules	Online Software	(80)
NetCTLpan	Combining predictions of proteasomal cleavage, transporter associated with antigen processing (TAP) transport efficiency, and MHC class I binding affinity to generate an MHC class I pathway likelihood score	Predicting for all MHC class I molecules with known protein sequence and allow predictions for 8-, 9-, 10-, and 11-mer peptides	Online Software	(81)
SYFPEITHI	Providing extensive MHC I and MHC II binding data	Predicting peptide binding	Database	(82)
MixMHCpred	Describing preferred peptide sequences established for each allele by a mixture model based on position weight matrices (pwms), and train on mass spectrometry elution data alone	Scoring different peptides and prioritizing the most likely hla-i ligands. As it is trained on naturally presented Peptides	Software	(83)
MHCflurry	Training the antigen processing model combining new models for MHC class I binding and antigen processing	predicting the comprehensive MHC class I presentation	Software	(84)
ProPred	Providing extensive MHC binding data	Predicting the ability of MHC class I and MHC class II molecules to bind peptides	Online Software	(85)
RANKPEP	Providing a position specific scoring matrix (PSSM) or profiling derived from a set of peptides known to bind to a specific MHC molecule	Predicting whether other peptides might bind and anticipating possible T-cell epitopes within a protein	Online Software	(86)
MaxQuant	For mass spectrometry (MS)-based analysis	Mainly using for proteomics research, but can also being used to help identify potential antigenic peptides in specific cases	Software	(87)
DeepNeo	Training models using deep learning algorithms combined with large-scale genomic and epigenomic data from tumor samples	Predicting the immunogenic neoantigen	Software	(88)

### 4.4 Immunogenicity of microbial peptides

Immunogenicity, as defined, encompasses the capacity of a molecule or substance to provoke an immune response, reflecting the intensity or magnitude of that response (94). When the innate immune system proves insufficient in fully eradicating a pathogen infection, the adaptive immune system, constituting the second line of defense, comes into play. In scenarios where specific antibodies have been generated from prior natural infections or vaccinations, the adaptive immune system is primed to mount a direct response upon encountering corresponding pathogens (95). An effective adaptive immune response hinges upon the precise pairing of an epitope with complementary T lymphocyte receptors (TcRs) (95). Importantly, immunogenicity and immunological memory are intimately linked. Immunological memory, a hallmark of adaptive immunity, can be engendered within the central or peripheral immune systems and stands as a primary outcome of successful specific responses (96). A robust immune response is typified by the sustained production of effector lymphocytes. Notably, the immunogenicity of an antigen inversely correlates with the quantity of antigen required to trigger an immune response. Consequently, heightened immunogenicity translates to reduced antigen demand for eliciting a response, fostering stronger peripheral memory and bolstering affinity in epitope or target interactions. In essence, the magnitude of immunogenicity profoundly influences the potency and duration of the immune response (95).

**Figure 3:**
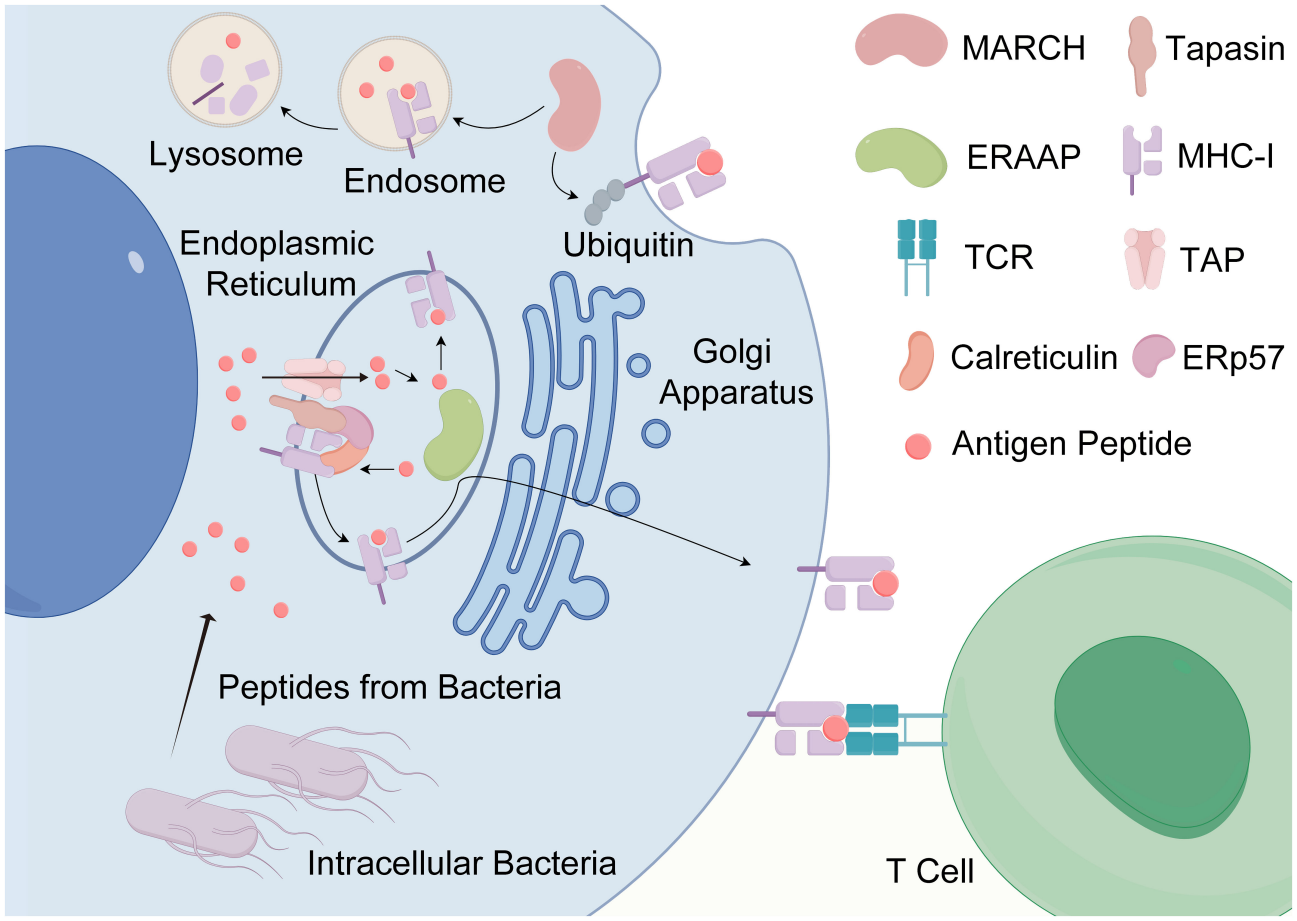
The antigen presentation of antigenic peptides. The antigen presentation of antigenic peptides, particularly those produced by intracellular bacteria, is a vital process in immune surveillance. Intracellular bacteria, being endogenous pathogens, generate highly homologous antigenic peptides that can be presented to T cells by any nucleated cell. The journey of peptides begins with their entry into the endoplasmic reticulum (ER) via the transporter associated with antigen processing (TAP). Within the ER, peptides undergo binding to MHC-I molecules with the aid of chaperones such as calreticulin and endoplasmic reticulum protein 57 (ERP57). Subsequently, these loaded peptides are shuttled to the Golgi apparatus and then transported to the cell surface for presentation to T cells. In addition, the process of antigen presentation can involve the internalization and degradation of MHC-I molecules in a ubiquitin-dependent manner. Some internalized MHC-I molecules may undergo recycling to the cell surface following exchange with endosomal peptides. Key regulatory proteins involved in this process include Membrane-Associated RING-CH protein (MARCH) and ER-associated aminopeptidases (ERAP) (created by Figdraw).

Indeed, the immunogenicity of antigens is governed by a multitude of factors, encompassing protein expression, antigen processing and presentation, peptide-MHC binding affinity, and the stability of resultant complexes, among others (97). Strong immune responses elicited by antigenic peptides underscore the efficient binding and presentation of these peptides by MHC molecules to active TcRs. In addition, the capacity of numerous MHC molecules to bind specific peptides suggests a relatively weak MHC restriction, permitting the presentation of these peptides to T cells by various MHC molecule types (95, 98). At the core of peptide immunogenicity lies antigen presentation, comprising the delivery of antigens to MHC molecules, loading of peptides onto MHC molecules, and subsequent display of MHC molecules at the cell surface (see **Figure 3**) (95, 98). The process is analogous to HLA molecules replacing the “housekeeping” peptide with a short amino acid sequence (8–20 aa) from the external antigen, aligning and complementing the antigen-binding groove on the HLA molecule (99). These amino acid sequences are securely anchored within the HLA binding groove through physicochemical interactions, facilitating engagement with corresponding TcRs on T lymphocytes. Subsequently, HLA molecules complexed with peptides are transported to the surface of antigen-presenting cells (APCs) via antigen presentation (95, 98). Moreover, the establishment of an optimal cellular milieu is imperative for fostering and sustaining the responsiveness of antigen-specific T cells and B cells. This involves epitope recognition and the interaction of specific TcRs with antigenic determinants. Furthermore, the balance between cytokines and other immune mediators considerably influences the outcomes of T cell activation and differentiation processes. Consequently, this intricate interplay can either amplify or attenuate the inherent immunogenicity of antigenic peptides (95, 100).

### 4.5 Validation of microbial peptide immunogenicity

To validate the activity of predicted microbial peptides *in vivo*, it is imperative to substantiate their immunogenicity through a series of rigorous laboratory experiments (8). Presently, *in vitro* validation procedures for immunogenicity encompass HLA binding assays and immunological analyses aimed at determining whether the selected peptides can be recognized by existing T cells. A crucial tool in this validation process is the Class I peptide binding assay, a classic competitive assay method. This assay relies on the inhibitory effect exerted by a specific radiolabeled peptide segment with high affinity, which competes for binding to purified MHC molecules harboring specific allele genes. Through this method, the quantitative measurement of the peptide’s binding capacity to MHC Class I molecules is achievable (101). In addition, the MHC Class I multimer binding assay plays a crucial role in elucidating whether particular peptide segments are capable of eliciting recognition by T cells via MHC Class I molecules. This assay is crucial for assessing the immunogenicity of peptide segments and their potential to stimulate immune responses (102–104). Moreover, the nanoparticle (NP) pull-down assay serves as a valuable technique in investigating protein interactions, particularly pertinent in the realms of immunology and cancer research. This assay facilitates the exploration of interactions between peptide-MHC I complexes and T cell receptors, providing insights into the immunological responses induced by the peptides (105). The selection of appropriate methods for immunogenicity validation hinges upon the specific objectives and requirements of the study at hand. For instance, in the study conducted by Wang et al., the researchers opted to employ an ELISpot assay coupled with T cell stimulation utilizing candidate peptides presented by dendritic cells. This methodological approach enabled them to gauge the specific T cell response triggered by the treatment regimen, thereby confirming the immunogenic activity of the peptides within the context of their study’s objectives (78). By employing these rigorous validation techniques, researchers can ascertain the immunogenicity of predicted microbial peptides, laying a solid foundation for their potential application *in vivo* and furthering our understanding of their therapeutic efficacy in cancer treatment.

### 4.6 Application of microbial antigen

Neoantigens, akin to bacterial antigenic peptides (**Table 2**) (49, 62, 63, 65, 77, 78, 106–111), emerge as promising candidates for tumor vaccines, capable of inciting tumor-specific immune responses. In mice, cross-reactivity between symbiotic bacteria and tumor antigens has been demonstrated to initiate anti-tumor responses (65). Specifically, CD8^+^ T cells have shown proficiency in recognizing peptides derived from *E. hirae* bacteriophage and tumor antigens through molecular mimicry cross-recognition (62). An approved therapeutic approach for bladder cancer involves the direct infusion of *Mycobacterium bovis* strain extract into the bladder, stimulating a helper T cell 1 (TH1) immune response from CD4^+^ T cells and imparting enduring protection in murine models (112, 113). Furthermore, Naghavian et al.’s findings illustrate that T cell clones TCC88 can directly target neoantigens in glioblastoma, exhibiting robust responses not only towards various glioblastoma-derived peptides but also towards targets originating from a diverse array of bacteria and intestinal microflora. These T cell clones elicit cross-reactive responses against tumor targets in numerous tumor-infiltrating lymphocytes (TIL) and peripheral blood memory T cells (77). Moreover, Wang et al.’s study suggests that the depletion of bacteria within tumors exposes bacterial epitopes, thereby precipitating immune responses aimed at eradicating tumor cells. In addition, the presence of homologous epitopes shared by bacteria and the host remarkably contributes to anti-tumor immunity. In a model of carcinogenic *F. nucleatum* infection, T cells exhibited responsiveness to epitopes shared by both *F. nucleatum* and the host, underscoring the potential of microbial epitope exposure in augmenting cancer treatment efficacy (78).

**Table 2:** The potential bacterial antigenic peptides for cancer therapy.

Disease Type	Host Species	Bacterial Species	Homologous Bacterial Peptides	Reference
Melanoma	Homo sapiens	*Bacillus polymyxa* *Escherichia coli*	GAGIGVLTA QAGIGILLA	(106)
Melanoma	Homo sapiens	*Chlamydia trachomatis*	MLSGIGIFFI	(107)
Melanoma	Homo sapiens	*Mycoplasma penetrans*	YIFAACLLLI	(108)
Pancreatic cancer	Homo sapiens	*Francisella tularensis*	KLLPEGYWV	(48)
Non-small-cell lung cancer	Homo sapiens	*bacteriophage Enterococcus hirae*	TSLARFANI KLAKFASVV	(62)
Melanoma	Mus musculus	*Bifidobacterium breve*	SVYRYYGL(SVY)	(65)
Melanoma	Homo sapiens	*Fusobacterium nucleatum* *Staphylococcus aureus* *Staphylococcus capitis*	ITELNSPVL, ITNTGAVTV, SLTDKISII, SVVVDELFEV, VLTDTYLTL EGNIDFITL, LSDLGKSIY AIGVAASILY, LEGTVLDTL, TPIVAVNAL, VTSGVTAAY	(63)
Colon cancer	Mus musculus	*Ruminococcaceae* *Bacteroidales/Prevotella/Muribaculacee* Bacteria_unclassified *Duncaniella/Bacteroides/Bacteroidales*	RLAGFFPRL LGPWRSGGVL SLPGSWRSL YIALFDGFI	(109)
Type 1 diabetes	Homo sapiens	*Parabacteroides distasonis 33B* *Bacteroides* sp.*CAG:144* *Ruminococcus gnavus CAG:126* *Coprococcus eutactus* *Clostridiales bacterium VE202–14* *Corynebacterium genitalium* *Lactobacillus vaginalis* *Lymphocystis disease virus 1* *Cyprinid herpesvirus 1* *Grouper iridovirus* *Burkholderia multivorans* *Bradyrhizobium japonicum SEMIA 5079* *Streptomyces griseus* *Tetrasphaera japonica T1-X7* *Saccharomonospora halophila* *Brevundimonas* sp.*BAL3* *Metarhizium robertsif*	RILVELLYLVCSEYL LDFKEALYLGCGDRT DPRRSALYLFCGKRC NHDKEALYIYCDETE VRAGYALFLVCDEEK FVHEDALHLVCGERI LOSMEIPYLVCGERE AHLVAALORVCGNRG SHPNVFIALVCGERG GELIDALTEHCGDRG LHLARALYEMCGEFP VSGKHALYLYCGERG RDRVEALRLVCGEAM HWLVEIAYLVCGDRR TAHGVAEYLVCGERR WVGFETLYLYCGERI DHWDEAGFLVCGERG	(110)
Autoimmune diseases	Homo sapiens	*Streptomyces Mobaraensis*	PSRMKAVIYSKHF NESAPAASSAGP APAASSAGPSFRAP REVASVMNRALE LCTAGFMPSAGEAAA AADNGAGEETKSY	(111)
Colorectal cancer	Mus musculus	*Fusobacterium nucleatum*	GVPQIEVTF, RQATKDAGTI, LADDNFSTIV, LADDNFSTI, RGVPQIEVTF	(78)
Glioblastoma	Homo sapiens	*Pectobacterium carotovorum* *Pectobacterium carotovorum* *Pectobacterium carotovorum* *Chlorobium phaeobacteroides* *Geobacter lovleyi* *Chlorobium phaeobacteroides* *Listeria welshimeri* *Chlamydia pneumoniae* *Chlamydia pneumoniae* *Chlamydia pneumoniae* *Geobacter lovleyi* *Chlamydia pneumoniae* *Pectobacterium carotovorum* *Buchnera aphidicola* *Geobacter lovleyi* *Streptomyces coelicolor* *Geobacter lovleyi* *Moorella thermoacetica*	TPILVDGKDVMPEVN TPILVDGKDVMPE NTPILVDGKDVMPE ALPVIETQAGDVSAYIP LNVLRIINEPTAAA LPVIETQAGDVSAYIP PAPATTFAHLD LQNIIPASTGAAK LQNIIPASTGAAKA NIIPASTGAAKA NVLRIINEPTAAA QNIIPASTGAAKA SNTPILVDGKDVMPE FRVPTANV GLNVLRIINEPTAAA DPAPATTFAHLDATTVLSR LNVLRIINEPTAA NPVDILTYVAWKISG	(77)

## 5 Conclusion and prospect

Tumor neoantigens have long been investigated as potential targets for cancer therapy, yet challenges persist regarding their applicability on a large scale and their efficacy against immune escape mechanisms induced by tumor mutations. Conversely, tumor-associated antigens, while prevalent across various malignancies, often elicit immune tolerance due to their expression in normal cells. Both neoantigens and tumor-associated antigens exhibit limited immunogenicity, highlighting the need for alternative approaches in cancer immunotherapy. Microbial antigen peptides, distinguished by their exogenous nature, possess robust immunogenicity, offering promising avenues for cancer treatment. Our review extensively explores the intricate interplay between microorganisms and tumorigenesis, shedding light on the immune mechanisms orchestrated by microbial antigens within the host immune system. By elucidating these mechanisms, we underscore the key role of microorganisms in tumor therapy and underscore the potential of microbial antigens in shaping the landscape of tumor immunotherapy. Studies have demonstrated that bacterial peptides with homologous epitopes to host antigens can induce potent immune responses, offering potential for anti-tumor therapy (63, 78).

Hence, the variation in intratumoral microorganisms dictates the presence of microbial antigens in a tumor-specific manner analogous to TAAs. For example, *Clostridium difficile* (*C. difficile*) is an important pathogenic bacterium that leads to colorectal cancer. Therefore, it is necessary to focus on *C. difficile* to explore microbial antigens for the prevention and treatment of colorectal cancer. Current research indicates that there are hundreds to thousands of different microbes present in tumors, but apart from *C. difficile* in colorectal cancer and human papilloma virus that can induce uterine cancer, there is still a lack of consensus on the major microbiota in other tumors. With the further study of microbiota in tumors, some specific bacteria are gradually being discovered. For example, in non-small cell lung cancer (NSCLC) tissues, the presence of specific taxa such as *Prevotella*, *Streptococcus* and *Veillonella* can lead to Phosphoinositide 3-kinase (PI3K) and protein kinase B(AKT) signaling activation (114). Pernigoni et al. found that specific *Ruminococcus* isolates were enriched in patients with castration-resistant prostate cancer (CRPC) and that these bacteria were able to synthesize dehydroepiandrosterone (DHEA) from pregnenolone, a precursor of testosterone (114). Developing bacterial antigens tailored to different tumor subtypes based on specific bacteria present in various tumors will be an important direction for identifying and characterizing bacterial antigens in the near future. Therefore, strategic integration of microbial antigen peptides into cancer therapy holds promise for enhancing immune responses against tumors.

We propose several avenues for leveraging microbial antigens in cancer therapy. Firstly, incorporation of microbial antigens into cancer vaccines or immunotherapeutic regimens can exploit their inherent immunogenicity to prime the immune system against cancer cells. In addition, the phenomenon of molecular mimicry between microbial and cancer antigens presents opportunities for developing personalized cancer immunotherapies based on individual microbial exposure histories. Moreover, therapeutic cancer vaccines incorporating microbial antigens offer potential for targeted cancer cell recognition and elimination, particularly when combined with tumor-specific antigens and advanced vaccine delivery systems. In practical clinical applications, we recommend using bacterial peptides identified by aligning the human reference genome or proteome with intratumoral bacteria for a more broad-spectrum cancer vaccine. These peptides can be encapsulated within diverse carriers, including liposomes, hydrogels, and engineered bacteria, and administered via routes like intramuscular and intravenous injections, as well as oral delivery. The process releases bacterial antigens, stimulating the generation of CD8^+^ T cells with anti-tumor capabilities in the body. For a more personalized treatment, identifying the bacterial species within the patient’s tumor through metagenomics and aligning them with the patient’s genome or proteome is necessary to select personalized bacterial homologous peptides. Furthermore, combination therapy approaches integrating microbial antigens with immune checkpoint inhibitors, chemotherapy, or other modalities hold promise for synergistically enhancing anti-tumor immune responses. By capitalizing on the complementary mechanisms of action of different agents, such combinatorial strategies offer potential for overcoming tumor immune evasion mechanisms and improving treatment outcomes.

Although microbial peptides have been recognized for their potential effects in tumor immune therapy, there are several challenges in clinical application. Of particular importance is the mutagenicity of tumors, which may hinder the long-term persistence of memory T cells stimulated by microbial antigen peptides, relegating them to a supporting role rather than a primary therapeutic modality. At second, the abundance of intratumoral microorganisms makes the screening for anti-tumor bacterial antigens even more challenging. Although computer-assisted screening has significantly narrowed down the range of peptides, the diversity of microorganisms means that each type of tumor will produce a vast array of microbial peptides. How to achieve high-throughput screening of tumor antigens will be a major challenge in developing anti-tumor microbial antigens. Therefore, in practical applications, it is crucial to have a sensible screening process, which may involve more peptidomics approaches. Thirdly, the initial screening of tumor antigens relies more on animal experiments, and the significant differences between animals and humans pose a risk of peptide failure in human bodies. Furthermore, despite some research findings from *in vitro* and *in vivo* animal experiments, there is still a lack of sufficient clinical studies, especially, to fully demonstrate the effectiveness of microbial antigens. Last but not least, if our prediction involves aligning the human reference genome with the homology of intratumor bacteria, it might lead to immune cells activated by microbial antigen peptides attacking normal body cells instead of specifically targeting tumor cells.

In conclusion, microbial antigens represent a promising frontier in cancer therapy, offering unique advantages in terms of immunogenicity, cross-reactivity, and versatility. Moving forward, concerted efforts are warranted to further elucidate the mechanisms underlying the anti-tumor immune response elicited by microbial antigens and optimize their integration into clinical practice. By harnessing the power of microbial antigens, we can advance towards more effective and personalized cancer treatments, thereby transforming the landscape of cancer therapy.

## Author contributions

TZ: Project administration, Software, Writing – original draft. XLZ: Data curation, Formal Analysis, Methodology, Validation, Writing – original draft. JC: Data curation, Formal analysis, Writing – original draft. XWZ: Conceptualization, Funding acquisition, Supervision, Writing – review & editing. YZ: Conceptualization, Funding acquisition, Supervision, Writing – review & editing.

## Glossary

NK cells: natural killer cells
TSAs: tumor-specific antigens
TAAs: tumor-associated antigens
MAGE: melanoma-associated antigen
ICI: immune checkpoint inhibitor
CAR-T: chimeric antigen receptor T cell
IL-2: interleukin-2
IFN-γ: interferon-gamma
PD-1/PD-L1: programmed death protein-1/programmed death ligand-1
Sag A: secreted antigen A
NOD2: nucleotide-binding oligomerization domain 2
DCs: dendritic cells
ILC: innate lymphocyte
GC cells: germinal center cells
MHC: histocompatibility complex class
PG: peptidoglycan
LPS: lipopolysaccharide
OMV: outer membrane vesicles
BG: bacterial ghosts
P-AKT: phospho-protein kinase B
P-mTOR: phospho-mammalian targets of rapamycin
P-p70s6K: phospho-p70 ribosomal s6 kinase
TMP1: tail tape measure protein 1
PSMB4: proteasome β subunit 4
PAMPs: pathogen-associated molecular patterns
Ags: Microbial antigens
HLA: human leukocyte antigen
IEDB: Immune Epitope Database
TcRs: T lymphocyte receptors
TAP: transporter associated with antigen processing
ERP57: endoplasmic reticulum protein 57
MARCH: Membrane-Associated RING-CH protein
ERAAP: ER-associated aminopeptidases
APCs: antigen-presenting cells
NP: nanoparticle
TH1: helper T cell 1
TIL: tumor-infiltrating lymphocytes
ANN: artificial neural networks
PWMs: position weight matrices
PSSM: position specific scoring matrix
MS: mass spectrometry
NSCLC: non-small cell lung cancer
PI3K: Phosphoinositide 3-kinase
AKT: protein kinase B
CRPC: castration-resistant prostate cancer
DHEA: dehydroepiandrosterone
